# The dawn of spatial omics

**DOI:** 10.1126/science.abq4964

**Published:** 2023-08-04

**Authors:** Dario Bressan, Giorgia Battistoni, Gregory J. Hannon

**Affiliations:** 1 CRUK Cambridge Institute, University of Cambridge, Li Ka Shing Centre, Robinson Way, Cambridge, CB2 0RE, United Kingdom

## Abstract

Spatial omics has been widely heralded as the new frontier in life sciences. This term encompasses a wide range of techniques that promise to transform many areas of biology and eventually revolutionize pathology by measuring physical tissue structure and molecular characteristics at the same time. Although the field came of age in the past 5 years, it still suffers from some growing pains: barriers to entry, robustness, unclear best practices for experimental design and analysis, and lack of standardization. In this Review, we present a systematic catalog of the different families of spatial omics technologies; highlight their principles, power, and limitations; and give some perspective and suggestions on the biggest challenges that lay ahead in this incredibly powerful — but still hard to navigate — landscape.

Nearly all of the seemingly infinite complexity of biology occurs in three dimensions. Although we are all familiar with studying the spatial architecture of proteins and macromolecular machines, studying the architecture of even simple organisms or single tissues requires not only deciphering the molecular profiles of thousands to millions of cells but also understanding how their spatial context influences their behavior. A recent burst of innovation in making multiplexed spatial molecular measurements is opening a new chapter in biological research and enabling a renewed embrace of the complexity of living systems. One of the first steps along this journey was the emergence of single-cell “omics” technologies that operate on disaggregated tissues. These methods enabled the discovery of new cell types (1), cast new light on organismal development (2), and launched the process of creating comprehensive catalogs of human and mouse tissues (3). However, biological processes happen in a spatial context, and the three-dimensional (3D) arrangement of cells in a tissue has a profound effect on their functions. For example, location constrains cell-cell interaction by modulating contact or short-range paracrine signaling, which creates specialized niches such as those that support many types of tissue stem cells. Spatial gradients of both physical and chemical properties also contribute to differential regulation of biological processes at every scale, but are particularly prominent during the development of multicellular organisms. Regardless of their undisputed power, measurements made on disaggregated cells or nuclei lack this layer of information. The need for such knowledge has driven the development of “spatial omics”: methods capable of measuring the molecular characteristics of cells in their native 3D context. Spatial omics technologies mainly measure either proteins and their modifications, or mRNAs, although spatial genomic and epigenomic profiling is now emerging. Compared with their nonspatial counterparts, these methods offer “shallower” profiling, often with an inverse relationship between the degree of spatial resolution (or profiling depth) and the experimental time investment. Despite these limitations, this nascent field has already provided substantial and varied insights into several fields, including animal development and brain structure (4–6) and features of the tumor microenvironment (TME) that correlate with patient outcome (7, 8). Although we are at the very beginning of the spatial omics revolution, progress is happening at breakneck speed, and it is clear that these approaches will drive us toward a much deeper understanding of biology in context.

## The current generation of spatial molecular profiling tools

The idea of performing spatial molecular measurements on tissue sections is hardly new. Immunohistochemistry (IHC) and in situ hybridization (ISH) have been in use for more than 50 years (9–11). Both ISH and IHC can be multiplexed to a degree corresponding to the number of dyes that can be differentiated during observation. However, a move toward “omic” level measurements by simply increasing the number of dyes is not possible. Spatial omics technologies therefore rely on other approaches. They may use nonmicroscopic methods such as mass spectrometry (MS) to detect analytes, perform cycles of staining and dye removal to achieve the required complexity, or harness the power of high-throughput DNA sequencing and barcoding.

## Multiplexed antibody profiling

**Figure 1:**
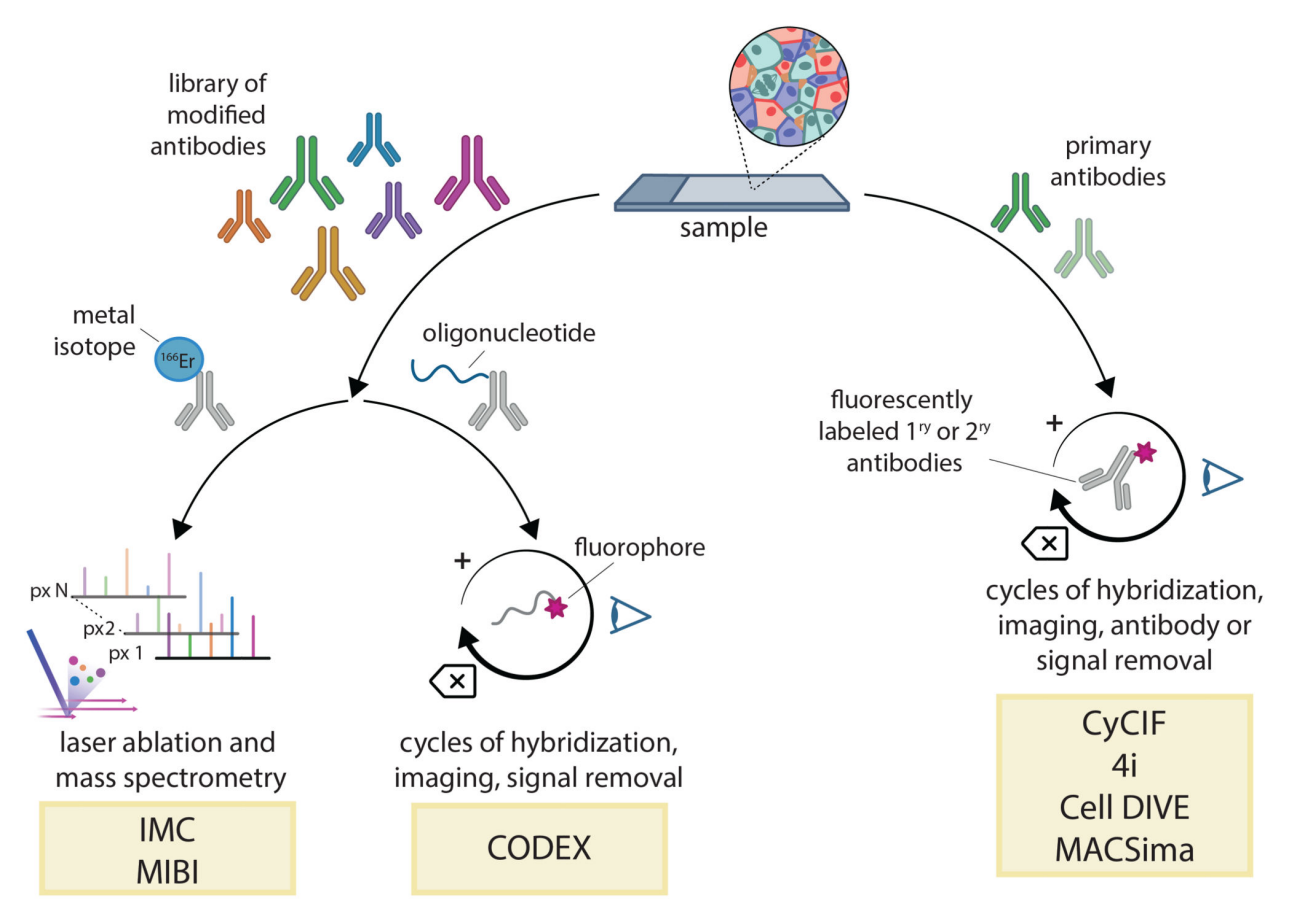
Spatial proteomics methods based on multiplexed antibody detection Proteomics methods can be broadly divided into two types of protocols. In one (left), antibodies are all bound together and detected either with MS imaging (IMC or MIBI) or cyclic fluorescence imaging (CODEX). In the other (right), primary antibodies are themselves bound and stripped in each cycle (4i, CyCIF, or Cell DIVE MACSima). In IMC and MIBI, each antibody is bound to a different metal by a chelating polymer. During imaging, a laser vaporizes the tissue pixel by pixel, and the metals released from each spot are quantified with a mass spectrometer. The distribution of metals can then be used to deduce the presence and spatial distribution of specific protein antigens in the tissue. The two methods differ in how mass measurements are performed and in their resolution: 1 µm for IMC and 300 nm for MIBI. In fluorescence-based methods, each cycle detects as many markers as there are florescence channels available, with complexity scaling linearly with the number of cycles. Binding all probes together (CODEX) results in faster imaging and shorter cycles but requires custom modification of each antibody and extensive optimization. On the other hand, binding antibodies in cycles (4i, CyCIF, MACSima, and Cell DIVE) enables the use of “native” antibodies that can be obtained commercially but results in slower cycles (up to 1 day per round). Owing to the repeated rounds of microscopy, all cyclic imaging methods are slower than IMC and MIBI on a per-sample basis and present more challenges for cycle-to-cycle alignment of images (registration). However, the data-generating capacity is actually higher because the slow element is the staining process, which can be performed in parallel on many samples, whereas the imaging itself is much faster than that with MS.

Over the past 20 years, MS has already been successfully adapted to in situ measurements (12). A more recent generation of MS, usually referred to as “mass cytometry” (MC), is based on detection of antibodies conjugated to isotopically pure lanthanide metals (Fig. 1) (13). Each antibody is bound to a different metal by a chelating polymer. During imaging, a laser vaporizes the tissue pixel by pixel. Because lanthanides are essentially absent from biological materials and their masses can be discriminated with great precision, this method allows the routine quantification of more than 40 different species with a very high signal-to-noise ratio (SNR). This approach is the basis of two spatial imaging technologies known as imaging mass cytometry (IMC) (14) and multiplexed ion beam imaging (MIBI) (15). Although initially developed to detect proteins and protein modifications through antibody binding, these methods have been adapted for RNA imaging by producing metal-conjugated hybridization probes (16). However, these approaches are limited by the number of discriminable masses and, more importantly, by the availability of sufficiently pure metals, a nontrivial issue considering the scarcity of some ores. Together, these factors constrain the achievable complexity to roughly 50 species.

Proteomics methods can be broadly divided into two types of protocols. In one (left), antibodies are all bound together and detected either with MS imaging (IMC or MIBI) or cyclic fluorescence imaging (CODEX). In the other (right), primary antibodies are themselves bound and stripped in each cycle (4i, CyCIF, or Cell DIVE MACSima). In IMC and MIBI, each antibody is bound to a different metal by a chelating polymer. During imaging, a laser vaporizes the tissue pixel by pixel, and the metals released from each spot are quantified with a mass spectrometer. The distribution of metals can then be used to deduce the presence and spatial distribution of specific protein antigens in the tissue. The two methods differ in how mass measurements are performed and in their resolution: 1 µm for IMC and 300 nm for MIBI. In fluorescence-based methods, each cycle detects as many markers as there are florescence channels available, with complexity scaling linearly with the number of cycles. Binding all probes together (CODEX) results in faster imaging and shorter cycles but requires custom modification of each antibody and extensive optimization. On the other hand, binding antibodies in cycles (4i, CyCIF, MACSima, and Cell DIVE) enables the use of “native” antibodies that can be obtained commercially but results in slower cycles (up to 1 day per round). Owing to the repeated rounds of microscopy, all cyclic imaging methods are slower than IMC and MIBI on a per-sample basis and present more challenges for cycle-to-cycle alignment of images (registration). However, the data-generating capacity is actually higher because the slow element is the staining process, which can be performed in parallel on many samples, whereas the imaging itself is much faster than that with MS. Despite this limitation, spatial MC methods have been extremely successful, in part owing to the early commercial availability of instruments and reagents. Hundreds of studies that used the technology have been published on a wide variety of subjects (8, 17–22). Immunology and cancer biology, in particular, have benefitted hugely from the ability to perform a detailed characterization of the TME. An example is a recent survey of approximately 500 human breast cancer samples, which detected specific TME motifs and indicated that spatial features correlate strongly with patient outcome (8).

An alternative method for multiplexed antibody imaging is based on detection of conventionally labeled fluorescent antibodies over repeated imaging cycles (Fig. 1). In some of these methods, all antibodies are bound to the sample at once, and detection is performed in cycles by secondary probes, typically fluorescent oligonucleotides hybridized or ligated to a “root” attached to the antibody [codetection by indexing (CODEX) (23)]. In others, antibodies are themselves bound in cycles and either stripped after each round of detection (24, 25) or made undetectable by means of photobleaching or chemical cleavage [CyCIF (26, 27), Leica Cell DIVE (28), and Miltenyi MACSima (29)]. Each of these strategies have distinct advantages and disadvantages in terms of cycling speed and antibody optimization (Fig. 1), but overall, they have a greater data-generating capacity relative to that of MS. Indeed, cyclic imaging methods are likely the most viable alternative for projects that involve large sample collections (30, 31).

## Spatial transcriptomics and genomics

The ultimate goal of a family of technologies known as “spatial transcriptomics” is to measure the abundance of every gene and gene isoform, at subcellular resolution, in a whole tissue sample in three dimensions. At present, no technology is able to reach this target. Instead, each protocol makes trade-offs in terms of sensitivity, throughput, resolution, or ease of use. Currently, all technologies work on histological sections of various thickness and can be largely considered “2D” methods, with some exceptions. Spatial genomic profiling is based on four main strategies: (i) microdissection methods in which specific areas of a tissue are physically isolated or otherwise marked so that their DNA or RNA can be extracted and processed; (ii) combinatorial fluorescence in situ hybridization (FISH) followed by single-molecule imaging, which allows counting of mRNA molecules present in a given area of a tissue; (iii) in situ sequencing of mRNA molecules; or (iv) spatial barcoding methods in which the DNA and mRNAs in a given area are linked to a specific predetermined DNA “barcode,” which allows them to be computationally mapped back to a spatial location after bulk sequencing. These approaches are described in more detail below.

### Microdissection and photo-isolation

**Figure 2:**
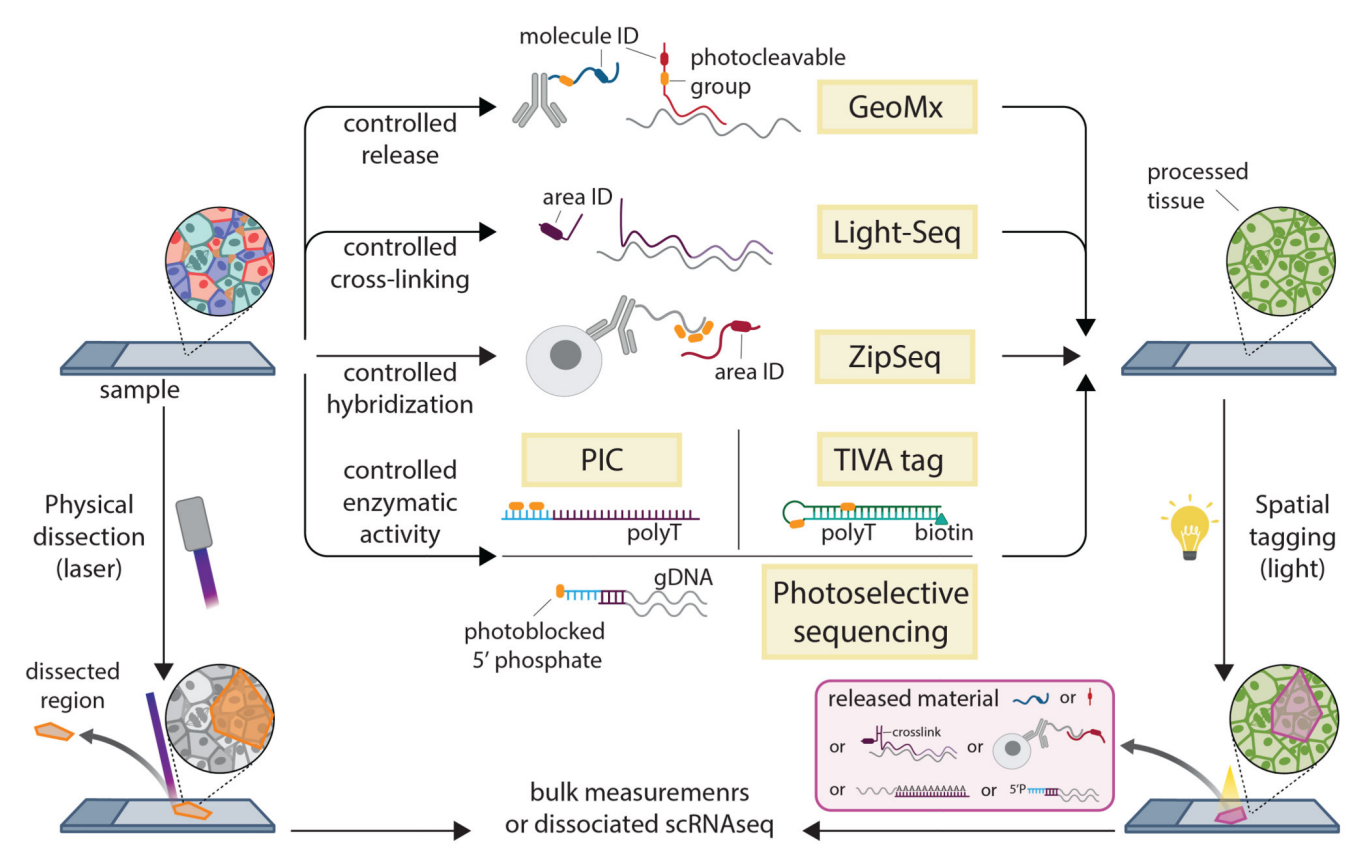
Spatial dissection and selective illumination methods. Several different methods can be used to restrict profiling to a specific section of a sample. Laser capture microdissection (LCM) relies on the physical isolation of a tissue fragment, from either flash-frozen or FFPE-treated material (24), followed by bulk measurements. By contrast, photo-sensitive groups can be used to tag an area of interest using light. TIVA-tag and PIC use photo-sensitive groups to trigger RT either in vivo or in fixed samples. Nanostring GeoMx uses the same cleavable group as that of TIVA to release a fluorescent combinatorial tag bound to detection probes (antibodies or hybridization probes), which is then quantified by using Nanostring’s nCounter technology. Light-Seq and ZipSeq use light tagging to assign an area ID by means of crosslinking or hybridization, respectively.

Microdissection is perhaps the most obvious way to add spatial information to molecular profiling (Fig. 2), through purification of biomolecules from a specific area by means of physical separation. Laser-capture microdissection (LCM) (32) is routinely used to isolate small areas (tens to hundreds of cells) for RNA-sequencing (RNA-seq) and can reach single-cell resolution (33).

Several different methods can be used to restrict profiling to a specific section of a sample. Laser capture microdissection (LCM) relies on the physical isolation of a tissue fragment, from either flash-frozen or FFPE-treated material (24), followed by bulk measurements. By contrast, photo-sensitive groups can be used to tag an area of interest using light. TIVA-tag and PIC use photo-sensitive groups to trigger RT either in vivo or in fixed samples. Nanostring GeoMx uses the same cleavable group as that of TIVA to release a fluorescent combinatorial tag bound to detection probes (antibodies or hybridization probes), which is then quantified by using Nanostring’s nCounter technology. Light-Seq and ZipSeq use light tagging to assign an area ID by means of crosslinking or hybridization, respectively. Recent studies have used light, rather than physical dissection, to spatially select a region to profile. Light can be used to trigger reverse transcription (RT) [transcriptome in vivo analysis (TIVA) tag or photo-isolation chemistry (PIC) (34, 35)], cleave a reporter [Nanostring GeoMx (36)], attach a purification label (Syncell MicroScoop), or remove a blocking group that prevents ligation of a sequencing adapter [photoselective sequencing (PSS)] (37). Light can also trigger attachment of a DNA barcode, or barcode combination, to specific areas either by driving their cross-linking or hybridization to the tissue (38,39). The barcode is then used to reassign reads to these areas. All microdissection technologies share some key advantages and drawbacks: They offer very deep profiling, almost to bulk-sequencing levels, and allow the area of interest to be customized from a single cell to a whole region. However, they have a comparatively low throughput (usually below hundreds of locations) because each selected area has to be collected and processed individually. Barcoding methods partially bypass this limitation but are still largely limited to hundreds of areas at most.

### Multiplexed ISH

**Figure 3:**
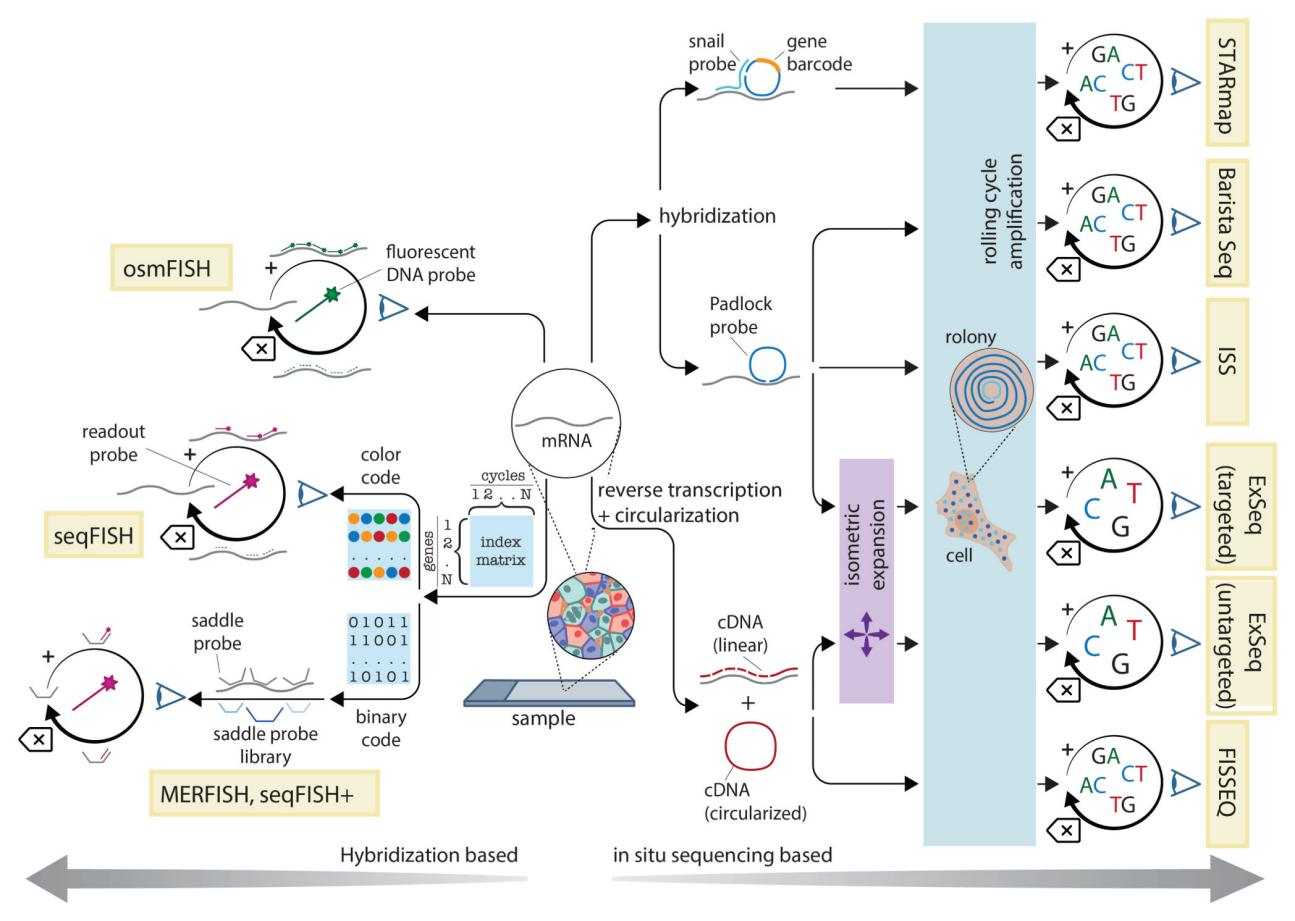
Imaging-based methods Molecules are detected through hybridization and identified according to patterns of fluorescence signal, either driven by cooperative hybridization or incorporation of fluorescent nucleotides. In osmFISH, transcripts are individually detected in separate cycles of hybridization and signal release. MERFISH, seqFISH, and seqFISH+ use combinatorial labeling schemes to assign each transcript to a barcode, consisting of a subset of hybridization probes that are detected in cycles of imaging and release. Each digit is read in a separate cycle, with different colors (seqFISH+) or presence and absence of fluorescence (MERFISH) corresponding to different values. This allows the detection of up to Xn genes (where X is the potential values of each digit and n is the number of cycles). Whereas seqFISH probes are directly conjugated to fluorophores, MERFISH and seqFISH+ use “saddle probles” with a region complementary to a set of fluorescently labeled “readout” probes, then are hybridized and read cyclically. Each spot corresponds to a RNA molecule, and the sequence of fluorescence signals identifies it. In “targeted” ISS methods, multiple transcripts of interest are bound by a library of detection probes that circularize through split ligation. ISS, BaristaSeq, and ExSeq use padlock probes to either form an exact junction or leave a gap that is filled by polymerase before ligation. In STARmap, a pair of oligonucleotides [snail (specific amplification of nucleic acids via intramolecular ligation element) probes] need to bind in close proximity to enable circularization. In “untargeted” methods, RNA is retrotranscribed from a poly(T) oligonucleotide, and the cDNA is then circularized through intramolecular single-strand ligation. The circularized molecules are amplified by means of RCA, producing rolonies. In all cases, sequencing is performed on the rolonies by using either sequencing-by-ligation or sequencing-by-synthesis technologies. Targeted methods read a barcode within the probe itsef or in the gap-filled region (allowing detection of mutations). Untargeted methods sequence the cDNA itself, which can be purified and sequenced again in bulk (ExSeq)

Cyclic hybridization–based methods (Fig. 3) are the descendants of single-molecule FISH (smFISH), a set of technologies that resolve individual mRNA molecules in tissues as subdiffraction fluorescent spots. To enable detection, these protocols require multiple probes to bind to the same mRNA molecule, either because multiple fluorophores bound to a small area are needed in order to produce a signal (40, 41) or because the concurrent binding of two probes within a few nucleotides of distance triggers a signal amplification process (42–44). smFISH is often considered the “gold standard” in RNA quantification methods because it can detect very low-abundance transcripts down to a single copy per cell, and therefore the spatial profiling technologies derived from it tend to have excellent sensitivity.

Molecules are detected through hybridization and identified according to patterns of fluorescence signal, either driven by cooperative hybridization or incorporation of fluorescent nucleotides. In osmFISH, transcripts are individually detected in separate cycles of hybridization and signal release. MERFISH, seqFISH, and seqFISH+ use combinatorial labeling schemes to assign each transcript to a barcode, consisting of a subset of hybridization probes that are detected in cycles of imaging and release. Each digit is read in a separate cycle, with different colors (seqFISH+) or presence and absence of fluorescence (MERFISH) corresponding to different values. This allows the detection of up to Xn genes (where X is the potential values of each digit and n is the number of cycles). Whereas seqFISH probes are directly conjugated to fluorophores, MERFISH and seqFISH+ use “saddle probles” with a region complementary to a set of fluorescently labeled “readout” probes, then are hybridized and read cyclically. Each spot corresponds to a RNA molecule, and the sequence of fluorescence signals identifies it. In “targeted” ISS methods, multiple transcripts of interest are bound by a library of detection probes that circularize through split ligation. ISS, BaristaSeq, and ExSeq use padlock probes to either form an exact junction or leave a gap that is filled by polymerase before ligation. In STARmap, a pair of oligonucleotides [snail (specific amplification of nucleic acids via intramolecular ligation element) probes] need to bind in close proximity to enable circularization. In “untargeted” methods, RNA is retrotranscribed from a poly(T) oligonucleotide, and the cDNA is then circularized through intramolecular single-strand ligation. The circularized molecules are amplified by means of RCA, producing rolonies. In all cases, sequencing is performed on the rolonies by using either sequencing-by-ligation or sequencing-by-synthesis technologies. Targeted methods read a barcode within the probe itsef or in the gap-filled region (allowing detection of mutations). Untargeted methods sequence the cDNA itself, which can be purified and sequenced again in bulk (ExSeq)

All cyclic FISH methods share some common elements: (i) a way to associate combinatorial barcodes to transcripts through the hybridization of short DNA probes; (ii) a method to read the barcodes, element-by-element, with cycles of fluorescence imaging; (iii) a strategy to produce sufficient signal on each molecule to enable detection, either through sheer probe number or by signal amplification; and (iv) a way to reset the signal after each cycle. In sequential FISH (seqFISH), fluorophore-labeled FISH probes are directly hybridized to cellular mRNAs and removed after each cycle through deoxyribonuclease (DNase) digestion, leaving the mRNA in place (45). In multiplexed error robust FISH (MERFISH), the probes include both a mRNA-binding region and a series of “reporter” regions that correspond to the barcode elements, which are detected with secondary hybridization rounds (46). This makes detection effectively independent of the original RNA molecule and makes the protocol more resistant to ribonuclease (RNase) contamination, while also decreasing imaging time and making imaging of hundreds to thousands of targets practical. An alternative version of seqFISH, seqFISH+, follows the same approach (47). Another element introduced by MERFISH and adopted in seqFISH+ is making the combinatorial barcode “error robust” by using encoding strategies taken from information theory, thus mitigating the impact of hybridization failures or nonspecific binding, which can be quite substantial owing to the cyclical nature of imaging.

In the past few years, both MERFISH and seqFISH+ have been extended to the whole-transcriptome level (47, 48), adapted to simultaneous detection of mRNA and protein by oligo-conjugated antibodies, and enhanced by the addition of signal amplification strategies and tissue embedding and clearing (44, 49, 50). MERFISH has also been adapted to expansion microscopy, a technique for physically expanding samples to increase the size of biological structures. Combining MERFISH with expansion microscopy (51) permits the identification of transcripts that would otherwise be too close to each other to be recognized as individual molecules. MERFISH and seqFISH+ are effectively very similar in terms of sensitivity, throughput, and pros and cons, with a few technical differences in protocol and details of the barcoding scheme. Both have been used successfully to investigate spatial gene expression in different organs and tissues, although their technical complexity has laborious implementation that has limited their broad adoption, although recently MERFISH has been made commercially available and simpler to use.

Although MERFISH and seqFISH are the two main hybridization-based in situ transcriptomic methods currently available, a few other technologies share some of their key elements. Ouroboros smFISH (osmFISH) is a cyclical smFISH method that lacks a barcoding scheme, trading multiplexing capacity (only a few transcripts per cycle are detected) for a simpler protocol not affected by transcript abundance or density (52). Saber-FISH and Clamp-FISH are based on different signal amplification methods and offer substantially improved SNR compared with that of “first-generation” protocols, but they have not been shown to enable detections of more than 100 transcripts (53–55). SCRINSHOT (56), a similar method, is specifically designed to detect approximately 30 transcripts on challenging formalin-fixed paraffin-embedded (FFPE) tissue. A more recent protocol called enhanced electric FISH (EEL-FISH) uses electrophoresis to drive cellular mRNAs to the surface of a conductive glass slide. The tissue is then removed, and the mRNAs are detected with multiplexed FISH (57). Removal of tissue results in a substantial increase in SNR and in much higher speed. Last, commercial options are becoming available for several of these technologies. Many of these solutions offer profiling of thousands of genes and, optionally, of tens of proteins simultaneously (58).

### In situ sequencing

Close relatives of cyclic-FISH protocols are in situ sequencing technologies (Fig. 3). The premise of this family of methods is to execute high-throughput sequencing chemistry on clusters generated in situ within a histological section. The sequencing technology used most often is sequencing by ligation (rather than sequencing by synthesis, which dominates traditional next-generation sequencing). A common step to all of these protocols is the production of a DNA “nanoball” (rolony) through rolling circle amplification (RCA) of a circular template produced from the detected transcripts. A separate rolony is produced for each mRNA molecule, allowing transcript quantification. The maximum read length is 30 nucleotides because long reads are much more difficult to perform in situ than on a flow cell owing to the many variables at play.

One of the first examples of these methods, ISS, is based on padlock DNA probes hybridized to mRNA (59–61). The padlock probe either produces an exact juxtaposition of the probe ends, which are joined by ligation, or leave a gap that is filled by a polymerase and then ligated. In the former variant, sequencing is performed on a reporter barcode sequence within the noncomplementary region of the probe, whereas in the latter, any sequence present in the gap is read. This allows both gene expression profiling (in which hybridization events to a preset list of genes are counted) or untargeted de novo sequencing of single-nucleotide polymorphisms or mutations (still limited to a specific gene list). The method evolved over time and was improved by replacing sequencing by ligation with a new form of sequencing by hybridization (in effect a form of specialized seqFISH-MERFISH performed on barcoded rolonies), with a resulting increase in SNR (62).

A contemporaneous technology called fluorescence in situ sequencing (FISSEQ) (63) generates the RCA amplicon through the circularization of a cDNA molecule, itself produced by means of RT of mRNAs by using an oligo(dT) primer. The technology allows true “untargeted” sequencing in space, although only for very short, 3’ terminal reads and at the expense of a very low sensitivity (well below 1% of total cellular transcripts) owing to the low efficiency of in situ RT and cDNA circularization.

ISS and FISSEQ rapidly gave rise to a variety of derivative methods with higher efficiency and improved features. In STARmap (spatially resolved transcript amplicon readout mapping) (64), two probes are used rather than a single padlock probe, one acting as a splint for the other upon binding in close proximity. This increases sensitivity, although still not in the range of the hybridization-based methods. The sequencing chemistry is also altered and improved compared with traditional sequencing by ligation. BaristaSeq (65) increases efficiency by optimizing the gap-filling padlock probe method and using Illumina sequencing by synthesis for detection [resolution is unclear because (65) generally applies the technique to the sequencing of a single barcode per cell]. More recently, both the targeted padlock-based ISS method and the untargeted FISSEQ method have been combined with expansion microscopy chemistry (66), with substantial improvements in both efficiency and SNR owing to the decrowding of rolony signals and sample clearing. One of the key innovations of ExSeq is the combination of untargeted, short-read ISS with regular “bulk” sequencing performed on the same sample after extraction of the cDNA contained in the tissue. The short sequences serve as distinct “tags” to match each “bulk” read to a specific location, effectively resulting in full-length, long-read ISS that is sufficient to map splice isoforms with subcellular resolution in complex tissues such as a mouse brain.

FISH-based and in situ sequencing-based methods share one common element: detection is ultimately performed by means of microscopy imaging of the tissue at high resolution, and gene expression is measured by effectively counting individual molecules of mRNA. Although imaging is the most direct way to obtain spatial information, single-molecule microscopy is very challenging and requires extremely precise instruments and procedures. This technical barrier is compounded by the need for multiple imaging cycles, combined with extensive sample manipulation through fluid exchanges, enzymatic reactions, or other procedures. Not surprisingly, image co-registration and feature extraction are among the biggest challenges in the analysis of all imaging-based spatial omics datasets (also discussed in Big data to useful data—the challenge of analysis). Single-molecule microscopy can also be slow because the need for high magnification and resolution produces a very small field of view, requiring extensive tiling and image stitching (in itself not a trivial procedure) to acquire any useful sample size. Molecular crowding within the cell also imposes an upper boundary to the number of molecules that can be resolved given the diffraction limit. Detection sensitivity is therefore biased by cell size, with larger cells allowing deeper profiling.

## Spatial barcoding of bulk sequencing reads

**Figure 4:**
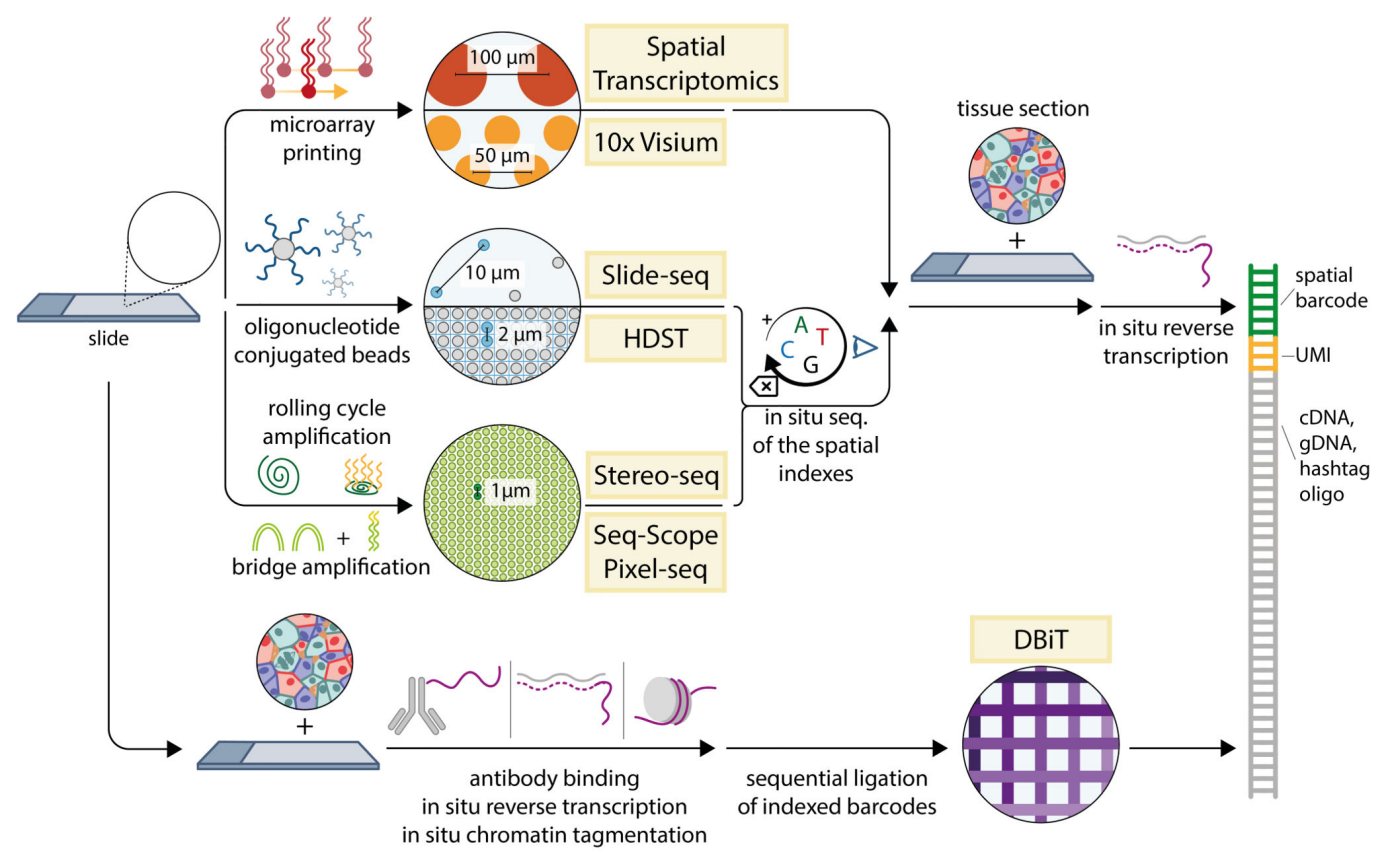
Spatial barcoding methods All methods are based on the production of a solid surface with a regular array of capture probes (DNA oligonucleotides), terminating with a poly(T) sequence that matches cellular mRNAs. In ST and 10X Visium, the capture probes are built directly by means of microarray printing, resulting in a resolution of 50 to 100 µm. In Slide-seq and HDST, the probes are synthesized on gel beads, which are then randomly attached to a slide or deposited on a microwell chip. The position of each bead is then detected by short rounds of ISS. In Seq-Scope and PIXEL-seq, the capture probes are produced by modifying sequencing clusters produced by bridge amplification on an Illumina flow cell or on a polyacrylamide-based hydrogel, whereas in Stereo-seq, they are produced by means of RCA on a silicon wafer. Spatial location is provided by ISS of the capture probes. In all cases, a tissue section is then placed on top of the arrayed capture probes and digested, letting the mRNAs in the tissue reach the poly(T) region, and cDNA is synthesized in situ. In DBiT-seq, barcodes are created directly on top of biomolecules (DNA fragments, cDNA, or oligo-conjugated antibodies) by placing a microfluidic chip with parallel thin channels on the section and performing ligation with a different oligonucleotide in each channel. The chip is then rotated by 90°, and ligation is repeated, producing an xy grid with resolution ranging from 10 to 50 µm. In all cases, the indexed biomolecules are then profiled in bulk by means of sequencing.

One might argue that the best available tool to produce high-quality transcriptome quantification data with very high speed and throughput is already available in the form of regular next-generation sequencing, if only spatial position could be encoded in sequence space. This is exactly the key insight behind ST (Fig. 4) (67). In ST, an ordered array of oligonucleotides is deposited on a glass slide by using microarray printing technologies. Each oligonucleotide includes a handle compatible with a downstream sequencing reaction, a barcode specific for each spatial position, a random UMI (unique molecule identifier) used to correct polymerase chain reaction (PCR) amplification bias, and a polythymidine [poly(T)] sequence used to capture polyadenylated [poly (A+)] messengers. Each array spot features a different spatial barcode. A thin histological section is then placed on the array, permeabilized to allow the cellular RNA to diffuse to the barcodeed oligos, and reverse-transcribed in situ to produce spatially indexed cDNAs. The latter are then amplified, adapter-ligated to produce a library, and sequenced by using standard next-generation sequencing. Some key advantages are the ability to produce long sequencing reads (although still biased for the 3’ end of transcripts), lack of reliance on complex imaging instruments, high speed (the time required for processing is effectively independent of sample size), and potential for parallelization. These features greatly facilitated commercial adoption of the technology. However, there are some notable drawbacks, including low resolution (currently 50 to 100 µm), a high cost per sample, and low efficiency of RNA capture (Table 1).

All methods are based on the production of a solid surface with a regular array of capture probes (DNA oligonucleotides), terminating with a poly(T) sequence that matches cellular mRNAs. In ST and 10X Visium, the capture probes are built directly by means of microarray printing, resulting in a resolution of 50 to 100 µm. In Slide-seq and HDST, the probes are synthesized on gel beads, which are then randomly attached to a slide or deposited on a microwell chip. The position of each bead is then detected by short rounds of ISS. In Seq-Scope and PIXEL-seq, the capture probes are produced by modifying sequencing clusters produced by bridge amplification on an Illumina flow cell or on a polyacrylamide-based hydrogel, whereas in Stereo-seq, they are produced by means of RCA on a silicon wafer. Spatial location is provided by ISS of the capture probes. In all cases, a tissue section is then placed on top of the arrayed capture probes and digested, letting the mRNAs in the tissue reach the poly(T) region, and cDNA is synthesized in situ. In DBiT-seq, barcodes are created directly on top of biomolecules (DNA fragments, cDNA, or oligo-conjugated antibodies) by placing a microfluidic chip with parallel thin channels on the section and performing ligation with a different oligonucleotide in each channel. The chip is then rotated by 90°, and ligation is repeated, producing an xy grid with resolution ranging from 10 to 50 µm. In all cases, the indexed biomolecules are then profiled in bulk by means of sequencing.

The resolution of ST was greatly improved a few years later by two methods, Slide-seq (68) and high-density ST (HDST) (69), which attach the barcoded poly(T) primers to nanobeads (which can have a much smaller diameter) rather than directly to the glass surface. The beads are then packed on an adhesive slide (Slide-seq) or on a microwell-patterned slide (HDST), reaching a much higher spatial density (10 µm for Slide-seq and 2 µm for HDST). Because the packing is random, the barcodes are first decoded spatially with a round of ISS, and the slides are subsequently used for a standard ST protocol. Compared with original ST, Slide-seq and HDST enable profiling at a resolution comparable with the size of single cells, but this comes at the cost of a more complicated protocol and reagent set up and a lower efficiency of RNA capture. A second version of Slide-seq pushed capture efficiency to the same neighborhood of disaggregated single-cell RNA-sequencing (scRNA-seq) (70). A more recent method from the same authors, Slide-tags, uses the same approach to add spatial addresses to single cells that are then processed by using regular disaggregated pipelines, enabling multi-omic spatial profiling with established techniques (71).

Another method, “Seq-Scope,” provides a substantial advance in the field by “double-dipping” into one of the most widely used next-generation sequencing platforms. In this approach, a modified sequencing flow cell is used to generate randomly distributed DNA barcodes by means of bridged PCR amplification. The clusters are then mapped with sequencing by synthesis, enzymatically processed, and extended to remove the distal sequencing handle and install a UMI and a poly(T) sequence (72). This effectively produces a very dense barcoded slide with a different spatial barcode every 500 nm, for which the spatial coordinates of each spot are already known. The slide is then used for a slightly modified ST protocol, and a second round of sequencing is used to determine RNA abundance. Two other approaches, Stereo-seq (spatial enhanced resolution omics sequencing) and PIXEL-seq (polony-indexed library-sequencing) use a carpet of DNA “nanoballs” growing on a slide to achieve similar submicrometer resolution. Both methods have a relatively high capture efficiency, on par or slightly above scRNA-seq (73, 74).

A coarser way to produce spatial barcodes, which is nevertheless very effective, uses a series of microfluidic channels pressed on top of a tissue sample to create a physical barrier. In DBIT-seq (deterministic barcoding in tissue for spatial omics sequencing), barcoded oligonucleotides flowing through each channel are ligated to target biological molecules, after which the microfluidic chip is rotated 90°, and the barcoding is performed a second time. This procedure produces a grid, in which each intersection has a different index. This method has been used to profile RNA (75), proteins (76), and chromatin features (77, 78), either alone or in combination (75, 79). Resolution varies between 10 and 50 µm depending on the chip size, with higher resolution being more technically challenging. Capture efficiency is quite high, likely because of the missing requirement of diffuse RNAs from the tissue onto a barcoded support (Table 1).

Spatial barcoding techniques combined with bulk sequencing have been very successful. The availability of commercial options, nonreliance of high-precision instruments, and compatibility with existent workflows has lowered the entry barrier for many groups. Data analysis is also comparably simple because all processing happens at the level of sequence data, an area of expertise more widespread in biological laboratories than image analysis. However, the low resolution and random positioning of the spatial barcodes, with respect to the sample, means that the information can sometimes average several cells of different types, confounding results. In spite of this, ST and its derived techniques have been successfully applied to many studies—in particular, in the fields of neuroscience and cancer biology (80–82)—and improved methods will presumably push adoption spatial profiling techniques even further.

## The balancing act of spatial profiling

In an ideal world, a spatial profiling method—whether it targets RNA, proteins, or DNA—would offer a near-complete and unbiased image of the entire molecular content of a cell, at subcellular resolution and in a short period of time, reliably and cheaply enough to be performed on many samples, including archival samples. All current technologies, however, entail certain compromises, and the choice of approach therefore depends on the specific needs of each particular study. Resolution, throughput, ease of implementation, robustness, and cost represent key variables in the spatial profiling balancing act (Table 1).

For protein profiling, the main decision is whether to invest in the IMC-MIBI ecosystem or opt for cyclic immunofluorescence protocols. IMC and MIBI are commercially available, robust, and (for IMC) widely used. Although antibody conjugation and validation can be time consuming, a growing catalog of preconjugated reagents is available, and there are many examples of successful studies that use this technology, including some large-scale ones (8,17–22). However, detection maxes out at fewer than 50 different markers, sensitivity is limited, and amplification methods are not yet available. Moreover, imaging time and cost are substantial, requiring a large investment in both instruments and reagents. Last, resolution is relatively coarse on IMC and generally insufficient to detect any subcellular structure beyond nuclei and membranes. MIBI offers a higher resolution, but at the cost of even slower processing.

Cyclic immunofluorescence has a much lower entry barrier because of its lower cost, and it offers higher throughput. It can detect more than 100 features given enough time and antibody availability. Moreover, some incarnations of the technology are compatible with native antibodies, reducing the need for optimization. Last, resolution is much higher, both in the xy and z dimensions (with confocal imaging options available). On the other hand, compared with MS, fluorescence imaging has a lower SNR, which makes some markers difficult to detect. Cyclical imaging also requires nontrivial data processing because multiple images need to be aligned, and tissue damage can become substantial after multiple cycles. Considerations are much more complex in the spatial transcriptomics area. If very high resolution is needed—for example, to resolve transcripts in different subcellular compartments—imaging-based methods (whether hybridization or sequencing based) are the clear winners. High resolution, however, makes them slow; imaging time scales with both sample area and number of target features (because more cycles are necessary), and the very small field of view necessitates extensive tiling, image post-processing, and registration.

Of the two subcategories, hybridization methods have better sensitivity but tend to produce weaker fluorescence signals (although amplification strategies are available) and may require reoptimization for different tissues owing to different optical properties. They also perform best with relatively thin sections (10 to 20 µm). In situ sequencing protocols, on the other hand, unvariably use signal amplification and produce stronger signals, require less optimization for different tissue types, and can image thicker samples (up to 100 µm or so). However, in situ sequencing exhibits the lowest sensitivity of all methods considered here, and when signal amplification is used to improve the SNR of hybridization methods, sensitivity tends to also decrease. This might not be a deal breaker because a sparse sampling of the transcriptome can still be informative as long as relative differences in expression are maintained. However, some of the most useful genetic markers (such as transcription factors) are expressed at very low levels and may be hard to detect with methods having low sensitivity. Untargeted profiling without the need for a predesigned probe panel is another advantage of in situ sequencing methods (especially ExSeq, which offers full-length reads), allowing for accurate quantification of splicing isoforms and mutations. These methods also offer whole-transcriptome profiling. The sensitivity of untargeted methods, however, tends to be lower than that of targeted ones because of the low efficiency of in situ RT. Also, recent hybridization-based methods effectively profile thousands of genes, enough to identify cell types, states, and pathway activity and extrapolate the expression of most of the remaining transcriptome (83).

Resolution and throughput are also critical parameters to consider for some barcoding-based methods. For the most part, these methods cannot associate a spatial barcode with a specific cell (because barcodes are randomly distributed on the slide relative to cell positions, and often overlap cell-cell boundaries). The most widely used technology at the moment, ST, has a low resolution limited to 20 to 100 cells per barcode, which makes it substantially harder to profile spatial neighborhoods in detail. Other protocols reach resolutions ranging from 10 µm per barcode (Slide-seq) to 500 nm per barcode (PIXEL-seq, Seq-Scope, and Stereo-seq), and microfluidics-based barcoding (DBIT-seq) also reach 10 µm. None of these solutions, however, is “cell aware”. Furthermore, sensitivity is relatively low (mainly because of the need for in situ RT), and cost per sample is generally higher than that of other methods. However, barcoding methods offer considerable advantages in terms of throughput because the acquisition time does not scale with either the sample size or the number of features detected, and multiple samples can be processed in parallel and sequenced together in bulk.

Last, laser capture dissection or spatial tagging methods such as TIVA or PIC should be considered when deep profiling of just a few spatial locations is needed. These methods are often simpler to implement and allow direct purification of selected material for analysis through a variety of bulk profiling methods.

## Big data to useful data—the challenge of analysis

Data acquisition is often assumed to be the most difficult step in spatial omics methods. However, data manipulation, analysis, and visualization are just as important if not more so. Spatial profiling methods generate such large datasets (often many terabytes), have influenced and biased by so many factors, that an experiment that costs thousands of dollars can be rendered completely useless without the appropriate analytical tools. The raw data are sometimes so large that they cannot be properly visualized without specialized hardware and software.

**Figure 5:**
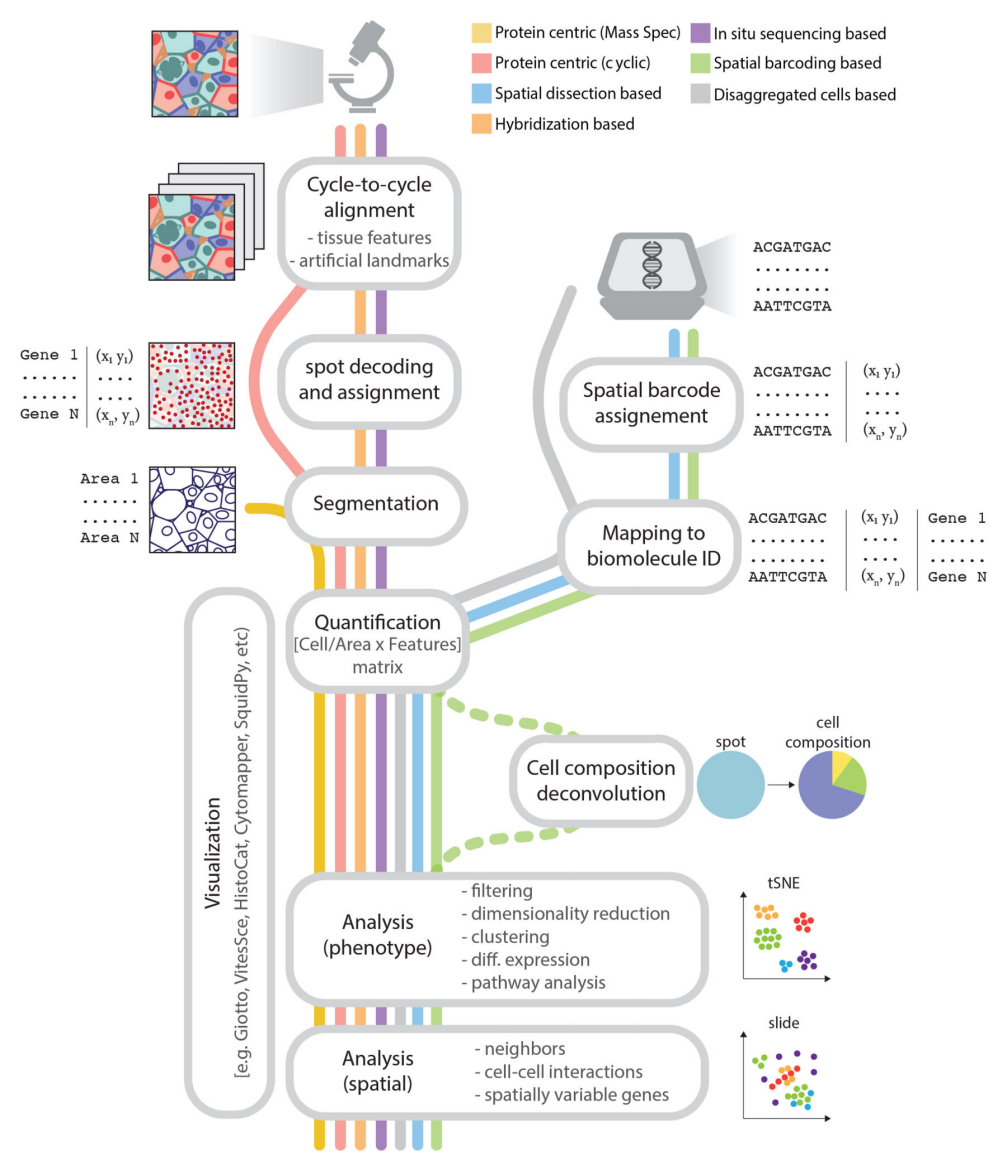
Analysis workflows for spatial profiling datasets Analysis workflows vary depending on the core acquisition modality (imaging or next-generation sequencing). Imaging methods require preprocessing of the images taken at different cycles and across multiple fields of view through stitching, background correction, and realignment with high accuracy (image registration). Image registration relies on easily identifiable “fiducial marks” that can be either point signals produced by the protocol itself (ExSeq and STARmap), embedded fluorescent microbeads (MERFISH and seqFISH), or the pattern of cell nuclei after intercalator staining (multiplexed IHC). For single-molecule imaging methods, the signal spots need to be identified and their patterns decoded to assign them to a biomolecule identification (ID) (usually a transcript). Images are then segmented to define areas corresponding to each cell (masks) by either identifying the cell membranes or performing a geometric expansion around the nucleus as a proxy for the cytoplasm. The number of spots or signal intensity are then integrated over the cellmasks, generating an area-by-features matrix. By contrast, spatial barcoding methods produce datasets in the form of sequencing output, which are preprocessed and parsed to assign each read to (i) a coordinate in space, through the location barcodes, and (ii) a biomolecule’s ID by mapping to a reference (such as a transcriptome). This is comparable with the preprocessing pipelines for disaggregated single-cell sequencing, with the space barcodes effectively replacing the cell barcodes, and representing areas that range from tissue regions to subcellular compartments, depending on the profiling method. Thereafter, common analysis steps include filtering, normalization, identification of highly variable features, dimensional reduction, clustering, and identification of differentially expressed markers. The results can be visualized either directly or in a dimensionality-reduced space on the basis of the omics profile and are finally integrated with the spatial information to reveal spatial features.

Some key steps of the analysis pipeline for each technology are illustrated in Fig. 5. Methods based on imaging face the immediate challenge of realigning images across the different image cycles and of stitching multiple fields of view into a coherent mosaic. Image registration is a nontrivial problem that has been extensively reviewed elsewhere (84, 85), made more complex in spatial profiling by the very large volume of data. Most methods resort to using easily identifiable “fiducial landmarks” (fluorescent beads) as features to calculate the registration matrix (Fig. 5), which make the registration much easier at the cost of additional experimental processing. In addition, most transcriptomic methods require a decoding step in which the sequence of fluorescent signals is matched to one of the genes in the panel, or resolved as a de novo sequence, and error correction and detection is applied. These processing pipelines need to be tailored to account for twists and aberrations specific to each technique or microscope and thus are difficult to standardize and optimize. Recently, the SpaceTx consortium made a substantial effort by releasing Starfish, a set of python modules successfully appliable to most methods (86), although generally underperforming each method’s dedicated pipelines.

Analysis workflows vary depending on the core acquisition modality (imaging or next-generation sequencing). Imaging methods require preprocessing of the images taken at different cycles and across multiple fields of view through stitching, background correction, and realignment with high accuracy (image registration). Image registration relies on easily identifiable “fiducial marks” that can be either point signals produced by the protocol itself (ExSeq and STARmap), embedded fluorescent microbeads (MERFISH and seqFISH), or the pattern of cell nuclei after intercalator staining (multiplexed IHC). For single-molecule imaging methods, the signal spots need to be identified and their patterns decoded to assign them to a biomolecule identification (ID) (usually a transcript). Images are then segmented to define areas corresponding to each cell (masks) by either identifying the cell membranes or performing a geometric expansion around the nucleus as a proxy for the cytoplasm. The number of spots or signal intensity are then integrated over the cell masks, generating an area-by-features matrix. By contrast, spatial barcoding methods produce datasets in the form of sequencing output, which are preprocessed and parsed to assign each read to (i) a coordinate in space, through the location barcodes, and (ii) a biomolecule’s ID by mapping to a reference (such as a transcriptome). This is comparable with the preprocessing pipelines for disaggregated single-cell sequencing, with the space barcodes effectively replacing the cell barcodes, and representing areas that range from tissue regions to subcellular compartments, depending on the profiling method. Thereafter, common analysis steps include filtering, normalization, identification of highly variable features, dimensional reduction, clustering, and identification of differentially expressed markers. The results can be visualized either directly or in a dimensionality-reduced space on the basis of the omics profile and are finally integrated with the spatial information to reveal spatial features.

Although most image-based protocols have subcellular resolution, almost all downstream analysis operates at the single-cell level. Once a molecule is detected in space, it needs to be assigned to a cell, and all the measurements taken over a cell area need to be integrated into a single abundance score for each gene or protein, ultimately creating a cell-by-feature matrix. To do this, raw images are segmented to identify areas that belong to individual cells.

Image segmentation is a central problem in both bioimaging and computer vision and has benefitted in the past few years from rapid advances in artificial intelligence (AI) and deep learning (87–89). Although nuclei segmentation benefits from the regular nuclear shape and the availability of a universal stain (DNA intercalators), to define cytoplasmic membrane boundaries is a more complex task. Membrane markers often generate low signal, have poor specificity for the cytoplasmic membrane, or are not generic for all cell types and thus cannot produce effective segmentation even when AI models are used. Although combining multiple labels has shown some good results (4), the development of a true universal membrane marker for segmentation would be transformative for the spatial omics field.

Spatial barcoding methods bypass most image analysis requirements because the data are in the same form as traditional sequencing methods, and it is already naturally matched to a specific location. Processing closely resembles genomics pipelines and is more standardized and portable, which represents a major advantage for this type of methodology. However, the “locations” to which barcoded reads are mapped do not necessarily correspond to specific single cells, and therefore the results are not a cell-by-feature matrix but correspond to “bins” of various size. In high-resolution methods such as Seq-Scope or PIXEL-seq, multiple bins can be summed to produce a cell-level database upon segmentation, which of course brings back the requirement for image analysis.

Once the initial processing is completed, the dataset is in a form comparable with that of disaggregated single-cell profiling methods, with the addition of spatial coordinates for each cell, and often with substantially higher cell counts. Tools designed for disaggregated data [such as the Seurat, ScateR, Scanpy, and Monocle packages (90–94)] can provide good results but need to be used cautiously because the nuances of data generation can cause biases. Some tools have been upgraded to handle spatial data, introducing data structures capable of holding coordinates and the ability to produce spatial plots and graphs. Dedicated packages have also begun to emerge, often integrating direct visualization of the raw data (for imaging-based methods) with analysis of the cell-by-feature matrix [for example, Giotto VitesSce, Histocat, Cytomapper, SquidPy, and others (95–99)]. Commercial options are also available.

Although standard dimensional reduction, clustering, and differential expression tools can operate effectively on spatial data, they fail to effectively leverage the most important feature of spatial profiling methods: space. The rapid development of spatial omics methods with ever-increasing resolution, throughput, and power has run ahead of the development of statistical frameworks and analysis tools capable of harnessing the data to generate new insights. This void is now starting to fill, with new algorithms being made available almost weekly. Although a review of this area would exceed the space available, and has been done in more detail elsewhere (100), there are three main areas of advancement: (i) identification of spatially variable markers and of specific expression patterns, through methods such as Gaussian process regression, spatial autocorrelation, and adversarial neural networks (101, 102); (ii) identification of spatial niches characterized by a set of coexpressed markers, at the level of multicellular areas or as a replacement or refinement for cell segmentation, under the assumption that cells could be defined as areas with a relatively uniform gene expression compared with other cells (103, 104); and (iii) identification of spatial interactions between different cell types (tissue neighborhoods), detecting whether two cell types are likely to colocalize in certain areas or conditions, and whether this correlates with tissue function or disease (105). This can be complemented by ligand-receptor analysis, a measurement of cell-cell functional interactions already introduced in disaggregated scRNA-seq (106–108), which really shines when combined with cell-cell proximity information.

## The challenge of experimental design

As the saying goes, “when you have a hammer, everything looks like a nail.” Spatial profiling technologies are wonderful new instruments, and it is tempting to apply them to every problem just because one can. To move forward, however, users will need to carefully formulate questions that can truly benefit from spatial omics and address them with experimental plans with a solid statistical foundation.

One application is the identification of new cell types and states within tissues, under both physiological and pathological conditions. This is the professed objective of the Human Cell Atlas project, which was initially focused on disaggregated methods but now is shifting to spatial omics technologies (3). These methods can reveal residual heterogeneity in gene or protein expression not accounted for by traditional cell-type definitions and correlate it with spatial location. Another appropriate question for these methods is whether colocalization of different cell types influences their expression profile or correlates with tissue function or disease outcome. This is the focus of several recent large-scale projects, investigating, for example, how tumors evolve and respond to treatment (17, 21, 22). The ability to simultaneously profile many genes and proteins also facilitates discovery of new markers for specific spatial niches. Because these technologies can produce detailed molecular profiling of tissues in their native state—without artefacts from dissociation, lysis, and averaging of different cells—they enable mechanistic investigation in situ of phenomena that could previously be approached only in simplified models or in a purely descriptive way.

Cell types, or states, characterized by a consistent marker profile can often be identified in spatial data by using the same tools used for disaggregated methods. These tools may be sufficient to define previously unknown populations even before location is considered. If the sample shows spatial consistency, cells of the same type can be manually pooled on the basis of prior knowledge of tissue anatomy—for example, comparing the abundance and expression profile of particular neurons in different cortical areas (5). If the spatial organization of the tissue is not consistent, a potential solution is to use spatial information to extract neighborhood scores or define “neighborhood clusters.” Scores reflect the frequency of contacts between different cell types, whereas clusters are assigned cells on the basis of the type of other cells they contact or their presence within a given range. These parameters are often directly comparable across samples even if the spatial structure itself is different. This approach has shown, for example, that colocalization of cells of different types in breast cancer has prognostic value (8).

One challenge in using spatial omics methods to characterize cell types is the limited number of features measured at each location. One strategy to overcome this limit is to combine spatial measurements with disaggregated single-cell methods, which often offer deeper profiling. The assumption behind such “data integration” is that a limited number of markers is sufficient to map each cell or location detected in the spatial method onto a cell class or state in the disaggregated data, and the additional measurements taken in the latter can then be reprojected in space. This approach has been used for both protein measurements, matching IMC and CyTOF (cytometry by time of flight) data (109), and RNA measurements (4). The latter case is particularly interesting because the authors were able to impute the values missing in the spatial data (in this case, a developing mouse embryo), producing a dataset that included more than 10,000 gene measurements per cell. Key to this integration is a well-chosen set of overlapping markers to join the two technologies. Currently, these are often chosen through manual curation or by identifying the most relevant cluster-specific features from disaggregated data. However, cluster-free a priori methods to identify optimal markers have recently been proposed (110). Notably, data integration is only possible for samples for which identical (or very similar) samples can be used for both disaggregated and spatial methods and in which both methods are likely to capture the whole heterogeneity of cell types and states present in the sample.

Replication is a central issue for much of the field, yet only possible if there is some degree of consistency in the spatial structure of samples. When studying tissues with a stereotypical organization, such as a developing embryo or the brain cortex, it is relatively simple to acquire multiple replicates and average the results. This is a challenge for pathological tissues (such as tumors) that have a highly heterogeneous, and not consistent, spatial structure. To further complicate things, as data resolution increases, sample-to-sample heterogeneity is revealed even in tissues with a seemingly repeatable structure, up to the extreme level of every sample being different, making replication impossible. This issue is already known to the field of disaggregated single-cell sequencing, which struggles with where to draw the line between one cell type or state and another.

In most cases, there is not yet a strong recommendation on the ideal number of replicates. Although published studies often use the “golden rule” of three or more replicates, a better option for large studies would be to first define the experimental parameters being compared, then begin with a small set of pilot experiments to estimate their variance across samples, and last, enroll the help of a trained statistician to calculate a sample size sufficient to produce a sound result. One must also consider the economic feasibility of big sample sizes. Application of spatial omics to large-scale projects is still hindered by the cost and time investment required by many technologies. This will undoubtedly be mitigated by further development and broader commercial diffusion of the technologies.

Studying rare or poorly characterized phenomena (such as micrometastases in distal organs, or rare or transient cell types in development) presents a different challenge: acquiring a sufficient number of samples. Because replication is a necessity, it is necessary in these cases to prescreen tissues with a limited number of relevant markers, ideally by using a fast, low-plex technology such as whole-slide scanning or 3D whole-organ microscopy. Areas of interest can thus be identified and prioritized for subsequent detailed molecular analysis. This has been done, for example, by using serial two-photon tomography (111, 112). The best experimental designs for spatial omics studies thus often include different but complementary technologies.

## Future directions

We are still at the dawn of the spatial omics era, and the future undoubtedly holds just as many revolutions and advancements as the past few years have seen.

Spatial profiling methods are going to become faster, more reliable, and more powerful with time. This will likely go hand in hand with a more extensive offer of commercial instruments and reagents. A handful of companies already offer “turnkey” solutions, with more set to launch. Commercial adoption in turn will facilitate wider access to these technologies and contribute to the democratization of spatial omics. Profiling depth is likely to continue increasing. Imaging-based RNA measurement methods have already reached whole-transcriptome levels, at least in cell culture, and this trend will likely extend to tissues. For proteins, whole-proteome measurements are presently unattainable, but the number of measurable markers will likely expand from less than 50 to hundreds, although this might require a move beyond MS-based methods and production of a large number of highly specific antibodies. Although ultradeep measurements will be possible, they will likely remain confined to special-use cases because garnering additional data must be balanced against increased experimental complexity. Untargeted in situ sequencing and spatial barcoding methods, which will no doubt improve in both specificity and resolution, will continue to represent a very valuable alternative, and the latter might well become the leading solution for large-scale studies.

We also anticipate a further extension of existing technologies to a wider range of measurements. Most existing methods profile either mRNA abundance or protein expression. More recently, RNA quantification has been achieved with multiplexed immunostaining methods that use fluorescent or metal conjugated probes (16), and conversely, oligonucleotide-conjugated antibodies have been used in both cyclic hybridization (48) and spatial barcoding methods (76).

Genomic and epigenomic measurements are also increasingly performed spatially. 3D chromatin organization has been profiled genome-wide, alongside gene expression, by using both seqFISH+ and MERFISH (113, 114), and the latter has also been combined with the Cut&Tag protocol (115) for spatial profiling of epigenetic marks (116). Spatial barcoding methods such as microfluidic barcoding and Slide-seq have been combined with both Cut&Tag and ATAC-seq (assay for transposase-accessible chromatin with high-throughput sequencing) (117) for spatial profiling of open chromatin and histone modifications (77, 78, 118). Last, detection of point mutations is possible by using ISS of DNA (119).

Although these different profiling modalities are still mostly performed one or two at a time (proteins + transcriptome, or transcriptome + genome organization), the future will unquestionably see a rise in spatial multi-omic technologies, mimicking the evolution of disaggregated single-cell technologies. Ultimately, a technology that incorporates genomic, transcriptomic, and proteomics–small-molecule measurements is desirable.

Last, the field will move closer to true 3D profiling. Currently, most spatial omics operate on thin tissue slices, which are essentially 2D. In the case of imaging-based profiling, the challenge is diffusing probes and antibodies through thick samples and obtaining sufficient SNR during fluorescence imaging. With barcoding methods, the oligonucleotides are placed in a 2D layer, and thick samples would result in multiple cells being captured for each position. Although 2D profiling is incredibly useful, it is blind to what lays outside of the sectioning plane and cannot produce an accurate image of some tissue niches. Only some multiplexed IHC and ISS techniques come close to 3D profiling (albeit only for samples a few hundreds of micrometers thick) because of the presence of strong amplification producing sufficient signal for confocal microscopy.

To enable 3D profiling more generally, one solution is to produce serial thin sections from a 3D sample, process each one through one of the 2D technologies, and use computational methods to realign the data and produce a 3D cube. This approach is very expensive in terms of processing time, but it is achievable with current technologies and has been demonstrated for both IMC alone, IMC combined with serial two-photon tomography, and spatial transcriptomics (111, 120, 121). Other approaches to 3D profiling will no doubt be developed in the coming years.

Just a few years ago, the ability to map the molecular make-up of every cell in a tissue, and interrogate hundreds of markers and millions of cells in the same experiment, would have been considered more science fiction than science. Now, single-cell and spatial omics technologies are advancing at breakneck speed and are granting scientists new powers to investigate complex biological processes. With this power comes the responsibility of assessing the limitations of this new breed of methods, their ideal application niches, and the best way to apply good experimental practices and rigorous analysis to the data that is produced. As more and more laboratories get access to these new-generation techniques, there is no doubt that incredible biological insights are just around the corner.

## Figures and Tables

**Table 1:** Summary of spatial omics technologies. IHC, immunohistochemistry; SNR, signal-to-noise ratio; IF, immunofluorescence; UMI, unique molecule identifier; FFPE, formalin-fixed paraffin-embedded; LCM, laser-capture microdissection; bin, 100 μm^2^ Rough cost ranges for instruments are defined as follows: high, > $500,000, medium: $100,000 to $500,000; low, <$100,000. Rough cost ranges per sample are defined as follows: high, >$1000; medium,: $100 to $1000; low: <$100. Numbers in parentheses refer to superscript numbers above them in their column, denoting the applicable methods.

	Multiplexed IHC/MS	Cyclic immunofluorescence	Microdissection	Light-based dissection	Multiplexed ISH	ISS	Spatial barcoding (patterned)	Spatial barcoding (patterned ligation)
Methods	IMC^1^, MIBI^2^	CyCIF^1^,4i^2^, CODEX^3^, MACSima^4^, Cell DIVE^5^, COMET^6^	Physical microdissection	PIC^1^, TIVA-tag^2^, PSS^3^, ZIPSeq^4^, Light-Seq^5^, GeoMx^6^, microSCOOP^7^	MERFISH^1^, seqFISH^2^, seqFISH+^3^, osmFISH^4^, SCRINSHOT^6^, EEL-FISH^6^	ISS^1^, FISSEQ^2^, ExSeq untargeted^3^ ExSeq targeted^4^ STARmap^5^, hybISS^6^	Visium^1^, SlideSeqV2^2^, Stereo-seq^3^, HDST^4^, Seq-Scope^5^, PIXEL-seq^6^	DBiT-seq
Modalities	Antibodies (1 and 2) and mRNAs (1)	Antibodies	Any downstream bulk method	RNA (1, 2, 4, and 5), DNA (3)	RNA (all), DNA (1 and 3)	RNA	RNA (all), DNA (2), antibodies (1)	RNA, DNA, Antibodies
Resolution	1 pm (1), 100s of nanometers (2)	Diffraction limited	Down to single-cell	Single-cell	Diffraction limited	Diffraction limited	<1 to 100 jim	Down to lOum
N. features	<50 (limited by availability of metal isotopes)	Up to 100s	Whole transcriptome or genome	Whole transcriptome or genome	Tens to ten thousands (cell culture)	100s to 1000s	Whole transcriptome or genome	Whole transcriptome or genome
N. areas	Scales with acquisition time, up to millions	Scales with acquisition time, up to millions	In practice, limited to tens to hundreds	Up to few hundreds	Scales with acquisition time, up to millions	Scales with acquisition time, up to millions	5000 to millions	2500
Sample area	Scales with acquisition time, up to centimeters	Scales with acquisition time, up to centimeters	Scales with acquisition time, up to centimeters	Scales with acquisition time, up to centimeters	Scales with acquisition time, up to centimeters	Scales with acquisition time, up to centimeters	4 mm to 2 centimeters	1 to 4 mm^2^
Sensitivity and capture efficiency	Up to standard IHC, excellent SNR and quantitation	As per standard IF, SNR affected by autofluorescence	As good as traditional RNA-seq or DNA-seq	Traditional RNA-seq, DNA-seq, or MS	Very high (single molecule per cell)	Varies: low, comparable, or better than that of scRNA-seq	−550 to 2000 UMI per bin	−2500 UMI per bin
Tissue	Frozen, FFPE	Frozen, FFPE	Frozen, FFPE (challenging)	Live (2 and 4), Frozen (all), FFPE (5, 6, and 7)	Frozen (all) FFPE (1 and 5)	Frozen (all), PFA-fixed (4)	Frozen (all), FFPE (1)	Frozen, FFPE
Commercial availability	SBT Hyperion (1), lonPath, MIBISCOPE (2)	Leica Cell DIVE (5), Akoya Phenocycler (3), Miltenyi MACSima (4), Lunaphore COMET (6)	Several suppliers	Nanostring GeoMX, Syncell MicroSCOOP	Vizgen MERSCOPE, 10X Xenium, Nanostring CosMX, Resolve biosciences, Rebus Esper		10X Visium, Curio Seeker	AtlasXomics
Cost (instrument/ per sample)	High/medium (antibody cost)	Medium/medium (antibody cost)	Medium/low (sample prep)	Medium/low (bulk sample prep)	Medium/medium	Medium to high/medium	Very low/medium to high (barcoded supports)	Low/medium
Required instruments	Dedicated MS	Standard fluorescence microscope or dedicated imager	Standard LCM instruments	Standard confocal microscope or other microscope with patterned illumination module	Dedicated epifluorescence or confocal microscope with fludiics	Dedicated confocal microscope with fluidics	No instrument (beyond NGS sequencer)	Facilities for microfluidic chip preparation (until commercially available)

## Structured Abstract

### Background

Just as single-cell sequencing has revolutionized many fields of biology, spatial “omics,” in which molecular parameters are measured in situ on intact tissue samples, is set to empower a new generation of scientific discoveries. A plethora of new technologies now enable spatial profiling of gene and protein expression, genetic mutations, epigenetic marks, chromatin structure, and genome organization. Most of these methods trace back to traditional techniques such as immunohistochemistry and in situ hybridization, or leverage the throughput of next-generation sequencing by converting spatial coordinates to sequence barcodes. Spatial omics technologies operate at vastly different levels of resolution, sensitivity, and throughput. There are also considerable differences in ease of adoption, compatibility with different sample types, commercial availability, upfront investment, and cost per sample. In such a rich and constantly evolving field, choosing the best technology to address a specific biological challenge—carefully weighting the strengths and weaknesses of each—is critical.

### Advances

The field of spatial molecular profiling has come of age over the past few years. Current RNA profiling technologies allow the spatial measurement of gene expression for centimeter-scale samples—in many cases, at single-cell resolution. Targeted methods can quantify thousands of transcripts, including those of very low abundance, whereas untargeted protocols offer transcriptome-wide profiling and can even detect gene isoforms and mutations, with coverage approaching that of disaggregated single-cell methods. Multiplexed immunohistochemistry protocols, either based on mass spectrometry or fluorescence imaging, can similarly be used to profile tens to hundreds of protein markers on a wide range of samples. Recent studies have reported additional approaches that recover other types of molecular information, including the spatial profiling of genomic organization for thousands of loci, open chromatin and epigenetic marks, and untargeted in situ DNA sequencing. Multi-omic technologies capturing multiple sources of information at once are also becoming available. As these methods become more reliable and widely adopted, it is becoming increasingly clear that spatial omics will contribute substantially to the elucidation of many biological questions, with prominent examples in fields ranging from oncology (such as the study of tumor heterogeneity and microenvironment) to neuroscience and organismal development.

### Outlook

Future development trends in the spatial omic area are needed in three main areas: (i) multi-omics, which is defined as the simultaneous measurement of different parameters (for example, DNA, RNA, and protein); (ii) increased access and democratization, with technologies becoming more readily available, more reliable, and more robust; and (iii) improved analysis frameworks. As technologies continue to evolve, it will become necessary to dedicate more attention to data analysis and experimental design. Spatial omics technologies can already generate many terabytes of data in a single experiment, creating substantial challenges in data processing, analysis, and visualization. Statistical methods and guidelines for sample replication, study design, and batch correction are also lagging behind, although they’re evolving rapidly. Last, as the future keeps getting closer, we foresee that spatial omics will likely evolve toward three-dimensional spatial omics (operating on whole organs or even organisms) and spatial-temporal omics (performing measurements multiple times in vivo), adding further powerful and exciting tools to biology’s toolbox.
